# Advance of Mesenchymal Stem Cells in Chronic End-Stage Liver Disease Control

**DOI:** 10.1155/2022/1526217

**Published:** 2022-10-07

**Authors:** Yun Gao, Xiushan Yin, Xiaomeng Ren

**Affiliations:** Applied Biology Laboratory, College of Pharmaceutical and Biological Engineering, Shenyang University of Chemical Technology, Shenyang 110142, China

## Abstract

The chronic liver diseases will slowly develop into liver fibrosis, cirrhosis, and even liver cancer if no proper control is performed with high efficiency. Up to now, the most effective treatment for end-stage liver diseases is liver transplantation. However, liver transplantation has the problems of donor deficiency, low matching rate, surgical complications, high cost, and immune rejection. These problems indicate that novel therapeutic strategies are urgently required. Mesenchymal stem cells (MSCs) are somatic stem cells with multidirectional differentiation potential and self-renewal ability. MSCs can secrete a large number of cytokines, chemokines, immunomodulatory molecules, and hepatotrophic factors, as well as produce extracellular vesicles. They alleviate liver diseases by differentiating to hepatocyte-like cells, immunomodulation, homing to the injured site, regulating cell ferroptosis, regulating cell autophagy, paracrine effects, and MSC-mitochondrial transfer. In this review, we focus on the main resources of MSCs, underlying therapeutic mechanisms, clinical applications, and efforts made to improve MSC-based cell therapy efficiency.

## 1. Introduction

Worldwide, approximately 2 million people die each year from liver diseases, accounting for 3.5% of all deaths [1]. Chronic alcohol abuse, virus infection, and autoimmune attacks stimulate hepatocyte apoptosis, endothelial barrier damage, inflammatory cell recruitment, and hepatic stellate cell (HSC) activation [2], resulting in liver fibrosis. Liver fibrosis slowly develops into liver cirrhosis, hepatocellular carcinoma (HCC), and eventually death from liver failure. Up to now, no effective treatment for end-stage liver diseases has been explored, except for liver transplantation. However, high costs, limited donors, and immune rejection after surgery limit the clinical utility of liver transplantation.

MSCs are mesoderm-derived multipotent stem cells with high self-renewal capacity and differentiation potential, which are widely found in a variety of tissues throughout the body. They can differentiate into mesodermal cell lineages and other germ layer lineages, including adipocytes, osteocytes, chondrocytes, and hepatocyte-like cells [3]. MSCs strongly express CD13, CD29, CD105, and CD44, weakly express CD106, do not express CD14, CD34, CD11a, CD31, CD45, and HLA II antigens, and neither express nor weakly express HLA I antigen [4]. MSCs have been demonstrated to be an effective therapeutic strategy in end-stage liver diseases due to their ability to transdifferentiate into the hepatocyte-like cells, immunomodulatory potential, paracrine activity, antioxidative capacity, derived extracellular vesicles, and regulation of cell ferroptosis and autophagy. This review will primarily focus on MSC resources, underlying therapeutic mechanisms, a summary of clinical applications, and several efforts made to improve MSC performance in treating end-stage liver diseases.

## 2. Main Resources of MSCs

**Figure 1:**
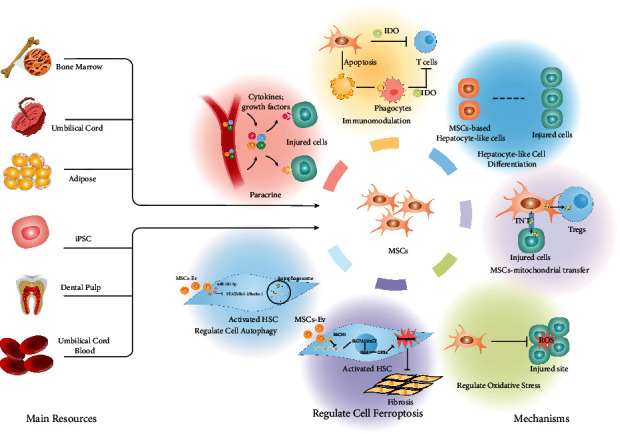
Main resources and underlying therapeutic mechanisms of MSCs in treating liver fibrosis/cirrhosis. MSCs are commonly obtained from bone marrow, umbilical cord blood, adipose tissue, and iPSC. MSCs exert therapeutic effects through paracrine, immunomodulation, hepatocyte-like cell differentiation, oxidative stress regulation, cell ferroptosis regulation, cell autophagy regulation, and MSCs-mitochondrial transfer.

MSCs are nonhematopoietic stem cells derived from the human mesoderm and widely distributed in the bone marrow, umbilical cord, adipose, and other tissues. MSCs were initially identified and isolated from bone marrow as adherent cells. But due to their limited numbers (0.01-0.001% of total bone marrow cells) [5] and the invasive isolation from bone marrow, researchers have explored other possible sources of MSCs. Several studies have reported the successful isolation of MSCs from various tissues with similar *in vitro* properties, including adipose tissue [6], umbilical cord [7], umbilical cord blood [8], synovium [9], amniotic fluid [10], and placenta [11], as shown in Figure 1. MSCs strongly express CD13, CD29, CD105, and CD44, weakly express CD106, do not express CD14, CD34, CD11a, CD31, CD45, and HLA II antigens, and neither express nor weakly express HLA I antigen [4]. The immunophenotypes of MSCs make it possible to be transplanted in an autologous or an allogeneic way, which broadens clinical applications. However, there are differences in the surface markers of MSCs due to the MSC sources [12], donor age [13], isolation methods [14], and culture conditions [15]. *In vitro*, MSCs of different origins differ in their ability to expand. Kern et al. found that bone marrow-derived MSCs (BMMSCs) had the lowest proliferation capacity and the shortest culture period, while umbilical cord-derived MSCs (UCMSCs) possessed the highest proliferation capacity and the longest culture period [16]. Similarly, MSCs from different sources exhibited different trilineage differentiation. Heo et al. found that UCMSCs can differentiate into adipose, bone, and cartilage and have a faster rate of osteogenesis with more ALP-positive cells and bone node formation under the same osteogenic conditions. UCMSCs and BMMSCs were both capable of differentiating into chondrocytes [17]. Besides, there were some differences in paracrine factor levels and immunomodulatory potential [18, 19].

### 2.1. Bone Marrow

In 1976, Friedenstein et al. found fibroblast precursors in the bone marrow, spleen, and thymus of adult mice, which could proliferate *in vitro* adherently, differentiate into other cell types, and showed colony growth capacity [20]. Subsequently, Pittenger et al. and Prockop found that fibroblast precursors contained MSCs, which could differentiate into multiple mesenchymal tissues, including bone, cartilage, adipose, and smooth muscle [3, 21]. Due to their linkage with the formation of mesenchymal tissues during embryonic development, these cells were termed “MSCs” [22]. Constrained by the limited number of BMMSCs and the invasive nature of harvesting, researchers have explored other possible sources of MSCs, like the umbilical cord [23], adipose tissue [24], and umbilical cord blood [17].

### 2.2. Umbilical Cord

In 1991, McElreavey et al. firstly isolated and characterized fibroblast-like cells from the Wharton's jelly portion of the human umbilical cord [7], which were found to differentiate into cartilage tissue when treated with TGF-*β* [25]. In 2005, Sarugaser et al. firstly isolated the nonhematopoietic human umbilical cord perivascular (UCPV) cell population. Human UCPV cells could expand rapidly and did not express MHC molecules, indicating that they could be applied for allogeneic mesenchymal cell-based therapies [26]. MSCs can be isolated from several compartments within the umbilical cord, including the umbilical vein, umbilical artery, and perivascular tissue of the umbilical cord, Wharton's jelly, and subamniotic tissue. Furthermore, UCMSCs are more primitive and abundant than MSCs isolated from other tissues. They are less likely to be contaminated by pathogenic sources and have a higher proliferation capacity than BMMSCs. UCMSCs have lower immunogenicity, no ethical controversy, and no harm to infants or mothers [17, 27].

### 2.3. Adipose

In 2002, Zuk et al. isolated MSCs from adipose tissue for the first time [28]. Adipose tissue is another source of MSCs. Obtaining adipose tissue is easier and less invasive than bone marrow, allowing it to be widely used [29]. Adipose-derived MSCs (ADMSCs) have multilineage differentiation potential and self-renewal capacity. The expression of stemness markers for different sources of MSCs showed the differences. BMMSCs highly expressed *SOX2*, *MYC*, *KLF4*, *NANOG*, and *INHBA* and did not express *OCT4*, *LIN28*, and *REX1*. ADMSCs highly expressed *MYC*, *KFL4*, *NANOG*, *LIN28*, *REX1*, and *INHBA* and did not express *OCT4* and *SOX2*. There were no significant differences between BMMSCs and ADMSCs in growth rate, colony-forming efficiency, and immunophenotype. BMMSCs and ADMSCs had the same trilineage differentiation capacity and gene expression profiles [17].

### 2.4. Pluripotent Stem Cell

In 2010, Lian et al. derived multipotent MSCs from human-induced pluripotent stem cells (iPSC-MSCs) [30]. iPSC-MSCs have been developed with a higher proliferation rate without loss of their key characteristics and engraftment capacity than other sources of MSCs [31, 32]. Furthermore, iPSC-MSCs had similar efficiencies with BMMSCs in homing to cancers but were much less prone than BMMSCs to promote the epithelial-mesenchymal transition, invasion, stemness, and proliferation of cancer cells [33]. Therefore, the *in vitro* differentiation of iPSC into MSCs may be proposed as a novel alternative resource, which helps to overcome several limitations of adult MSCs as seen in BMMSCs. Induction of glutathione peroxidase 3 (GPx3), which is reduced in senescence, from iPSC-MSCs can effectively control ischemia-reperfusion-induced liver injury via alleviating hepatic senescence [34] The limitations of adult MSCs sometimes were discussed as follows: iPSC-MSCs show a better competence in expansion, preserving differentiation capacity and proper karyotypes for 120 doublings, while BMMSCs become senescent after 20 doublings [30]. Besides, when isolated from elderly subjects or patients with age-related disorders, BMMSCs may exhibit reduced survival and differentiation ability, and iPSC-MSCs may overcome the aging-associated impairment of BMMSCs. This might be significant given that age is a factor in the prevalence, morbidity, and mortality of some diseases [35]. iPSC-MSCs also secreted abundant factors which can suppress inflammatory-like stem cell factor (SCF) [35], monocyte chemotactic protein 1 (MCP) [36], TGF-*β*1/2/3 [33], and FGF21 [37]. The consistency, quality control, and sufficient quantity have always been challenges of MSCs in clinical trials and medication development. iPSC-MSCs turned out to be effective solutions to solve these problems [38].

## 3. Underlying Mechanism of MSCs in Treating End-Stage Liver Diseases

MSCs have several potential regulatory mechanisms for the treatment of liver disease, including paracrine secretion, immune regulation, hepatocyte-like cell differentiation, antioxidation, regulation of cell ferroptosis, regulation of cell autophagy, and MSC-mitochondrial transfer, as shown in Figure 1.

### 3.1. Paracrine

MSCs have been shown to induce liver repair, ameliorate systemic inflammation, promote angiogenesis, and inhibit cell death and fibrosis through paracrine effects. Paracrine action is based on the secretion of cytokines, chemokines, trophic factors, and extracellular vesicles (EVs) [39–41]. This part focuses on MSC-derived EVs and nutritional molecules.

MSC-derived EVs contain exosomes (Exs). Exs are membrane-derived nanoscale vesicles that carry large amounts of proteins, nucleic acids, lipids, and metabolites that can be released and taken up by most cells [42]. MSC-derived Exs (MSC-Exs) have received a lot of attention as the most important potential cell-free therapeutic approach for liver diseases [43]. Exs derived from human UCMSCs (HUCMSC-Exs) reduced collagen deposition and oxidative stress in the liver, inhibited intrahepatic inflammatory cell infiltration, hepatocyte apoptosis, and liver structural damage, and improved CCl4-induced mouse liver fibrosis [44]. Moreover, HUCMSC-Exs significantly improved liver function, inactivated the TGF-*β*1/Smad signaling pathway, and inhibited EMT, which is a physiological process during liver fibrosis [45]. Human BMMSC-derived Exs (HBMMSC-Exs) repaired the liver structure and decreased fibrous capsules, collagen fibers, and lipid peroxidation changes in the rat liver intoxicated with CCl4. Besides, HBMMSC-Exs alleviated liver inflammation, improved liver function, and promoted liver regeneration. Mechanically, those Exs suppressed HSC activation through the Wnt/*β*-catenin signaling pathway [46].

MSCs can also secrete a large number of liver nutritional factors, which are constituents of the MSC secretome, to promote hepatocyte proliferation, reprogram HSCs, and enhance angiogenesis, such as hepatocyte growth factor (HGF) [47], nerve growth factor (NGF) [48], epidermal growth factor (EGF) [49], transforming growth factor (TGF) [47], and insulin-like growth factor-1 (IGF-1) [50]. HSCs play an important role in liver fibrosis pathogenesis. Thus, blocking HSC proliferation and enhancing HSC apoptosis can be a promising treatment for liver fibrosis. MSCs secreted HGF and TGF-3, which increased p21 and p27 while decreasing cyclinD1, leading to HSCs G (0)/G (1) arrest eventually [47]. MSC-derived HGF induced activated HSC apoptosis. Neutralizing HGF exhibited a weakened proapoptotic effect [51]. Besides, MSCs produced NGF, which promoted HSC apoptosis via NF-*κ*B and B cell leukemia-xl (Bcl-xl) molecules [48]. Milk fat globule-EGF factor 8 (MFGE8), one of the soluble proteins from the UCMSC secretome, significantly downregulated HSC activation by reducing the TGF-*β*1 receptor of HSCs [49]. Three-dimensional- (3D-) cultured ADMSCs, which produced high levels of HGF, IGF-1, SDF-1, and other proteins, showed a protective effect on liver fibrosis [50].

### 3.2. Immunomodulation

As an immune organ, the liver has a variety of immune cells, including infiltrating monocyte-derived macrophages, Kupffer cells, neutrophils, dendritic cells (DCs), natural killer (NK) cells, T cells, and B cells. Multiple immune cells secrete cytokines, chemokines, and other inflammatory factors to maintain liver homeostasis [52]. MSCs act as regulators for a variety of immune cells [53]. For end-stage liver diseases in animal models and clinical trials, MSCs secrete various immunosuppressive soluble mediators to inhibit T cell proliferation and activation, including IL-10 [27, 54] and indoleamine 2,3-dioxygenase (IDO) [55]. MSCs or MSC-conditioned medium (MSC-CM) treatment increased IL-10 and IDO levels in serum and ameliorated CCl4-induced liver fibrosis through downregulating IL-17 producing CD4+ T cells and upregulating IL-10 producing CD4+ T cells in the liver. Moreover, IDO inhibitor decreased the capacity of MSC-CM. Thus, IDO secreted by MSCs regulated liver fibrosis [55]. In steatohepatitis-induced liver fibrosis, ADMSCs restored liver function, improved parenchymal cell regeneration, and ameliorated liver fibrosis. ADMSCs decreased the ratio of CD8+/CD4+ T cells, which was consistent with the downregulation of antigen presentation and helper T-cell activation genes [56]. Primary biliary cirrhosis (PBC) is one of the most common types of liver cirrhosis. BMMSC transplantation significantly increased CD4+ Foxp3+ regulatory T cell levels in peripheral blood and in lymph nodes. Besides, BMMSCs reduced IFN-*γ* and elevated TGF-*β*1 in serum without affecting IL-10 expression [54]. From clinical trials, we can better understand the immunomodulatory role of MSCs. In a clinical trial for chronic hepatitis B-induced decompensated liver cirrhosis, human UCMSC administration reduced TNF-*α* and IL-6 levels markedly in serum compared to the control group [27]. Besides, human UCMSCs accelerated IL-10 production. CD4+ T cells and regulatory T cells were higher in the human UCMSC treatment group, while CD8+ T cells and B cells were much lower than in the control group [27]. For UDCA-resistant PBC, BMMSC transplantation improved liver function. In peripheral lymphocytic subsets, CD8+ T cells decreased, while CD4+ CD25+ Foxp3+ T cells increased. Besides, IL-10 levels in serum elevated [57]. BMMSC administration for HBV-related liver cirrhosis markedly improved regulatory T cells and reduced Th17 cells compared to the control group. Serum TGF-*β* levels were elevated, while IL-17, IL-6, and TNF-*α* were lower in the transplantation group than in the control [58]. All the data showed that MSC treatment exhibited immunomodulatory effects in the regulation of liver cirrhosis.

### 3.3. Hepatocyte-Like Cell Differentiation

Hepatocytes undergo apoptosis [59], necrosis [60], and pyroptosis [61] in the liver fibrosis/cirrhosis, resulting in the loss of normal hepatocytes and the damage of parenchymal structure and function. MSCs have the potential to differentiate into hepatocyte-like cells, making it possible to reconstruct the parenchyma of the liver [62]. Numerous approaches can promote MSCs transdifferentiating into hepatocyte-like cells [62]. Supplementation with growth factors, cytokines [63–65], and other compounds, like Chinese medicine [66, 67], promoted the conversion of MSCs to hepatocyte-like cells, alleviating liver fibrosis and cirrhosis. Genetic modification enhanced the differentiation of MSCs to hepatocyte-like cells and promoted liver function recovery in animal liver cirrhosis models [68]. In addition, changing the culture microenvironment of MSCs affected the differentiation capacity. MSCs expressed liver-specific genes, like *albumin*, *CK-18*, *CK-19*, and *AFP* when cocultured with liver cells [69]. 3D culture maintains adult hepatocyte function and the maturation of hepatic progenitors [70]. MSCs cultured in cell pellets and cell pellets supplemented with extracellular matrix (ECM) induced hepatic differentiation of MSCs [71]. In the preclinical experiments, MSC transplantation to the fibrotic or cirrhotic liver stimulated the production of hepatocyte-like cells and facilitated the repair of liver function [72, 73].

### 3.4. Antioxidative Capacity

Excessive ROS production exceeds the scavenging capacity of antioxidants in end-stage liver diseases, causing oxidative stress and hastening the pathogenesis of liver fibrosis or cirrhosis [60]. Clearing large amounts of ROS and improving antioxidant performance can be a promising strategy for end-stage liver disease treatment. BMMSCs showed antioxidative activity in end-stage liver diseases. BMMSCs migrated into injured sites in liver fibrosis animal models [74, 75], significantly reduced ROS production [76], downregulated lipid peroxidation (LPO) [75], improved SOD activity [76, 77], and increased GSH [74, 75] and antioxidant enzyme levels via Nrf2/HO-1 signaling pathway [75, 78]. In addition to BMMSCs, human amniotic membrane-derived MSCs significantly reduced oxidative stress in decompensated liver cirrhosis [79].

### 3.5. MSCs Regulate Cell Ferroptosis

Ferroptosis is one kind of programmed cell death different from apoptosis, necrosis, and autophagy, which is iron-dependent [80] and related to the pathogenesis of many diseases, like Parkinson's syndrome [81], cancers [82], and liver diseases [83]. Human UCMSC-Exs induced human hepatic stellate cell line ferroptosis *in vitro* without affecting hepatocyte ferroptosis. Mechanically, human UCMSC-Exs-derived BECN1 induced LX2 ferroptosis by reducing system xc-/GPX4 activity. In contrast, the knockdown of *BECN1* weakened the effect of human UCMSC-Exs on LX2 ferroptosis [84]. The data demonstrate that MSCs ameliorate end-stage liver diseases via modulating liver cell ferroptosis.

### 3.6. MSCs Regulate Cell Autophagy

The delivery of cytoplasmic cargo to the lysosome for degradation is the original scientific definition of autophagy. There are three forms of autophagy up to now, which are chaperone-mediated autophagy, microautophagy, and macroautophagy [85]. Autophagy plays an important role in the preservation of cellular and organism homeostasis [86]. ADMSCs delivered miRNAs to HSCs. ADMSCs overexpressed miR-181-5p communicated with HST-T6 cells mediated by Exs. Exs of miR-181-5p induced HST-T6 cell autophagy via downregulating the STAT3/Bcl-2/Beclin 1 signaling pathway *in vitro*. Furthermore, ADMSC-Exs of miR-181-5p attenuated liver fibrosis *in vitro* and in CCl4-induced liver fibrosis of mice [87]. In CCl4-induced liver fibrosis, circRNA (mmu_circ_0000623) was reduced compared to wild-type mice. Interestingly, ADMSC-Exs overexpressed mmu_circ_0000623 markedly inhibited CCl4-induced liver fibrosis by activating miR-125/ATG4D-mediated autophagy. However, the autophagy inhibitor reversed the autophagy activation effects resulting from Exs [88]. Some other sources of MSCs, like tonsil-derived MSCs (TMSCs), ameliorated liver fibrosis in mice by mediating autophagy. After TMSC treatment, autophagy-related proteins were detectable in parenchymal cells, and TGF-*β*, which is a marker of liver fibrosis, was not observed. Autophagy inhibitor, bafilomycin A1, suppressed the therapeutic effects of TMSCs [89]. All these findings indicate that MSC-mediated autophagy in the liver is a possible mechanism for MSC treatment of liver fibrosis.

### 3.7. MSC-Mitochondrial Transfer

Mitochondria of mesenchymal stem cells are involved in immune regulation [90–94]. Nonalcoholic fatty liver disease (NAFLD) will further develop into nonalcoholic steatohepatitis (NASH) [95]. NASH is pathologically defined by steatosis, inflammation, hepatocellular damage, and varying degrees of liver fibrosis [96, 97]. BMMSCs transfer alleviated (NASH) induced central carbon, amino acid, and lipid metabolism impairment related to mitochondrial and peroxisomal functional disorders. MSCs reduced fat load in hepatocytes by transferring mitochondria into hepatocytes via tunneling nanotubes (TNTs) [90]. Regulatory T cells (Tregs) have crucial functions in the maintenance of peripheral tolerance, the prevention of autoimmune illnesses, and the limitation of chronic inflammatory diseases [98]. Tregs inhibited inflammation in end-stage liver diseases. Hepatic Tregs downregulated severity of liver fibrosis in CCl4-induced chronic liver inflammation. Depletion of Tregs aggravated inflammation and fibrosis [99]. Direct and indirect contact with allogeneic ADMSCs improved therapeutic potential of Tregs via enhancing immunosuppressive adenosine accumulation and inhibiting the proliferation of conventional T cells. Especially, direct communication between ADMSCs and Tregs was achieved by transferring mitochondria and fragments of plasma membrane to Tregs via direct ADMSCs-Tregs contact in an HLA-dependent way [92]. Mitochondrial transfer from MSCs to human CD3+ T cells increased T cell activation and Treg differentiation-related mRNA expression compared to CD3+ T cells without mitochondria from MSCs, including FoxP3, CTLA4, and GITR. Functional analyses also revealed that transfer of mitochondria induced Treg cell differentiation and increased immunosuppressive efficiency [94]. Although there are few studies on the effect of mitochondrial transfer from MSCs on the activation of regulatory T cells in the treatment of liver fibrosis, this is a promising therapeutic strategy.

iPSC-MSCs as an essential source of MSCs showed impressive therapeutic effects on inflammatory diseases [91, 100–108]. Chronic obstructive pulmonary disease (COPD) exhibits severe fibrosis with mitochondrial dysfunction [93]. Human iPSC-MSCs or adult BMMSC transplantation showed protective effects against cigarette smoke- (CS-) induced lung damage via alleviating linear intercept and severity of fibrosis. MSCs transferred mitochondria to rat airway epithelial cells in lung sections exposed to CS. Furthermore, mitochondrial transfer from iPSC-MSCs to bronchial epithelial cells performed better than BMMSCs, with adenosine triphosphate content preserved. This unique mitochondrial translocation was facilitated by tunneling nanotube (TNT) production. Mitochondrial transfer was stopped when the formation of TNTs was blocked [93]. Similarly, higher expression of intrinsic Rho GTPase 1 (MIRO1) in human iPSC-MSCs than in BMMSCs was responsible for the greater efficiency of mitochondrial translocation in alleviating anthracycline-induced cardiomyocyte (CM) damage. TNF-*α*/NF-*κ*B/TNF*α*IP2 signaling pathway was responsible for TNT formation for mitochondrial transfer to CMs [91]. Mitochondrial transfer from iPSC-MSCs to epithelial cells in asthma rescued mitochondrial dysfunction in epithelial cells and downregulated asthma inflammation in vivo via TNT formation between iPSC-MSCs and epithelial cells [101]. Mitochondrial dysfunction was also observed in hypoxia-ischemia-induced brain injury. In vitro, TNTs were formed between iPSC-MSCs and PC12, a kind of neural cell lines. PC12 damage was relieved by transfer of mitochondria via TNTs. Damage to the channel reduced the effectiveness of the protection [108]. These data showed prospects for cell-based therapies for end-stage liver disease.

## 4. Clinical Application of MSCs in the Treatment of Chronic End-Stage Liver Diseases

**Figure 2:**
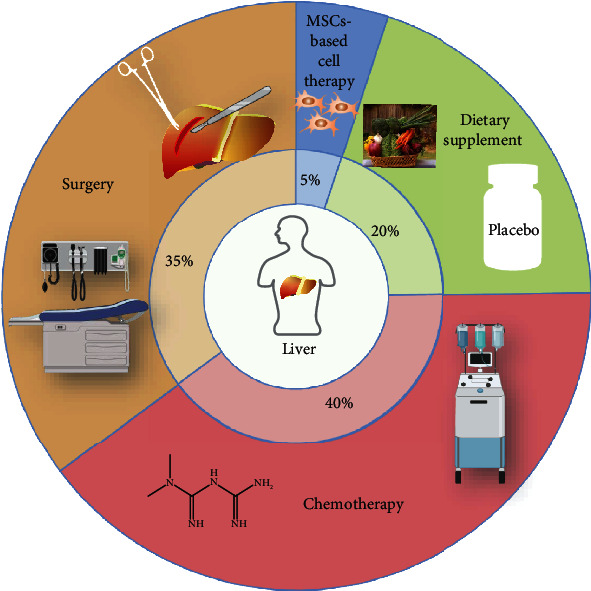
Treatments for end-stage liver diseases. The proportion of MSC-based cell therapy clinical trials compared with other conventional trials in liver fibrosis/cirrhosis.

In recent years, MSCs have brought a new dawn to treating various significant diseases, and related clinical research has been carried out in many countries. To date, approximately 11529 MSCs studies are documented on clinicaltrials.gov (*https://clinicaltrials.gov*). They mainly treat graft-versus-host disease (GVHD), hematopoietic system diseases, liver fibrosis/cirrhosis, diabetes, autoimmune diseases, and neurodegenerative diseases. 4019 studies have been completed, and most clinical phase II trials have yielded positive results, demonstrating that MSC-based therapy has broad application potential [23, 109, 110]. The liver is a vital organ with detoxification and regenerative capacities. However, viruses, alcohol, and other factors chronically damage hepatocytes and the endothelial barrier, inducing inflammatory cell infiltration, leading to massive production of collagen and extracellular matrix accumulation by activated HSCs, resulting in liver fibrosis, which will progress to cirrhosis in advanced stages. Liver failure occurs when the damage exceeds the compensatory capacity of the liver. Currently, the most effective treatment for liver failure is orthotopic liver transplantation. However, due to the shortage of donor organs, high costs, and the need for lifelong immunosuppressive drugs, liver transplantation cannot be used widely. According to *https://clinicaltrials.gov*, traditional treatments for chronic end-stage liver diseases include dietary supplementation, chemotherapy, and surgery. Due to the shortcomings of traditional treatment methods, MSC-based therapy gains attention as a potential therapeutic approach, as shown in Figure 2. There are 14 clinical trials in PubMed searching for “liver cirrhosis” and “mesenchymal stem cell”, which are listed in Table 1. Focusing on ongoing or completed clinical trials of MSCs in liver fibrosis/cirrhosis, which are registered at *https://clinicaltrials.gov*, approximately 68 studies demonstrated the popularity of MSCs. We listed the top 29 most recent studies with the highest correlation, as shown in Table 2.

## 5. Efforts to Improve the Therapeutic Performance of MSCs

### 5.1. Gene-Modified MSCs

Modification of MSCs using genetic engineering technologies can improve therapeutic outcomes for liver diseases [123]. Viral vector-mediated gene modification provides a potential new approach for remodeling MSCs, including lentiviruses, adenoviruses, and retroviruses, which enables MSCs to express a variety of exogenous genes with high expression and hardly affects the biological characteristics of MSCs [124]. Genes transfected into MSCs are classified as MMPs [125], microRNAs [87, 126], trophical factors [63, 127–130], transcription factors [131, 132], and ECM protein [133].

Matrix metalloproteinases (MMPs) take charge of degrading ECM [134]. MMP-1 was transduced into BMMSCs by a recombinant adenovirus vector. BMMSCs which overexpressed MMP-1 alleviated CCl4-induced liver fibrosis and liver injury [125]. MSC-Exs contain numerous microRNAs, which regulate intracellular signaling pathways [135]. *In vitro* assays revealed that ADMSCs overexpressing miR-181-5p built cell communication with HST-T6. miR-181-5p inhibited HST-T6 activation and promoted HST-T6 autophagy via direct targeting of Bcl-2 and STAT3. Moreover, ADMSCs-Exs containing miR-181-5p alleviated CCl4-induced liver fibrosis *in vitro* and *in vivo* [87]. Similarly, miR-122-modified ADMSCs by lentivirus significantly blocked HSC proliferation and collagen maturation. Exs produced by MSCs released miR-122 and facilitated communication between ADMSCs and HSCs. miR-122-modified ADMSCs showed better therapeutic effects against CCl4-induced liver fibrosis [126]. Erythropoietin (EPO), a glycoprotein hormone, is mainly produced by the kidney and can be found in other tissues, exhibiting anti-inflammation [136], neuroprotective [137], antioxidative stress [138], and antiapoptosis functions [139]. EPO modification enhanced BMMSC viability and migration ability. EPO-modified BMMSCs increased therapeutic efficacy towards liver fibrosis in an animal model. Besides, EPO elevated cytokines released from BMMSCs against liver injury [127]. HGF plays an important role in hepatocyte regeneration [140]. Besides, HGF can inhibit tissue fibrosis and apoptosis [141–143]. HGF modification improved the migration capacity of ADMSCs to injury sites. In addition, HGF-modified ADMSCs alleviated radiation-induced liver fibrosis and promoted liver regeneration as well as liver function [128]. Human UCMSCs modified with HGF improved liver function and recovered body weight and liver weight, thereby attenuating CCl4-induced liver fibrosis in rats [129]. IGF-1 is an essential hormone in metabolism, which is mainly produced by the liver. IGF-1 was downregulated in liver cirrhosis [144]. Modifying BMMSCs with the *IGF-1* gene did not affect the immunogenicity of MSCs. Furthermore, IGF-1-modified BMMSCs mitigated liver fibrosis in mice through downregulation of HSC activation. IGF-1-modified BMMSCs-CM elevated HGF transcriptional levels in hepatocytes [130]. Fibroblast growth factors (FGFs) regulate cell proliferation, differentiation, and migration [145]. Among FGF family members, FGF4 possesses the highest mitogenic activity in vitro [146–148]. FGF4-modified BMMSCs showed enhanced stemness. Moreover, FGF4 promoted BMMSC migration to the cirrhotic sites of the liver, leading to the improvement of hepatocyte and hepatic progenitor cell (HPC) proliferation [63]. Smad7 is a negative regulator of the TGF-*β*1/Smad signaling pathway, which is required for the pathogenesis of liver fibrosis [149]. BMMSCs transduced with the *Smad7* gene via a lentivirus vector exerted therapeutic effects by reducing fibrosis biomarkers, such as collagen I and III, TGF-*β*1, *α*SMA, TGF-*β*1R, and TIMP-1 [131]. Hepatocyte nuclear factor-4*α* (HNF-4*α*), as a transcription factor, regulates mature liver cell marker expression and plays a key role in liver cell maturation [150, 151]. Overexpressing HGF-4*α* in BMMSCs ameliorated CCl4-induced hepatocyte necrosis and fibrosis, and enhanced liver injury repair without affecting the homing of MSCs. Besides, HGF-4*α*-modified BMMSCs downregulated Kupffer cell activation and promoted iNOS expression via the NF-*κ*B signaling pathway, thus reducing liver inflammation [132]. ECM1, which is secreted by hepatocytes, regulates hepatic homeostasis. ECM1 is identified as a differentially expressed gene in liver cirrhosis, which is downregulated under cirrhotic conditions [133]. ECM1 expression was reduced during fibrotic pathogenesis in the liver, while ECM1 supplementation prevented fibrosis [152]. Hair follicle-derived MSCs (HFMSCs) were genetically modified with lentivirus to overexpress ECM1. ECM1-modified HFMSCs blocked the activation of HSCs via inhibiting the TGF-*β*/Smad signaling pathway *in vitro* and *in vivo*. Besides, ECM1-modified HFMSCs migrated to the injury sites of the liver and expressed hepatocyte-specific surface markers, thus ameliorating liver fibrosis and promoting liver repair and enhancing liver function [133]. All data suggest that genetic modification of MSCs is beneficial in enhancing therapeutic effects for liver fibrosis.

### 5.2. Pretreatment of MSCs

Due to the severe oxidative stress microenvironment in end-stage liver diseases, preincubation of MSCs with antioxidants improves the effectiveness of MSCs [153]. Melatonin (MT) is an endogenous neurohormone produced mainly by the pineal gland that exerts immunomodulatory, anti-inflammatory, cytoprotective, and antioxidant effects [154]. Similar to BMMSCs without preincubation, MT-pretreated BMMSCs (MT-BMMSCs) showed protective effects against liver fibrosis. MT-BMMSCs, on the other hand, migrated to injury sites in the liver more than nontreated BMMSCs [155]. Another study showed that MT-BMMSCs performed better than nontreated MSCs, especially in elevating glycogen storage, downregulating liver fibrosis, and reversing liver function [156]. Vitamin E pretreatment of Wharton's jelly-derived MSCs (Vit E-WJMSCs) ameliorated CCl4-induced hepatocyte injury *in vitro*. Furthermore, Vit E-WJMSCs recovered CCl4-induced liver fibrosis in rats and increased homing of WJMSCs [153]. *In vitro*, GSH or MT preconditioning improved ADMSC survival and migration. GSH or MT pretreatment of ADMSCs (GSH-ADMSCs or MT-ADMSCs) enhanced cell engraftment in the injury sites *in vivo* and restored live function in liver fibrosis [157]. Icariin (ICA), a traditional Chinese medicine, promoted the migration of UCMSCs towards the damaged liver tissue. Furthermore, ICA-treated UCMSCs accelerated liver function recovery from CCl4 intoxication, reduced oxidative stress, and blocked the progression of liver fibrosis in mice [158]. Enhancement of MSC-converted hepatocyte-like cells is a strategy to improve the therapeutic effect. MSCs pretreated with injured liver tissue increased the expression levels of hepatocyte markers compared to normal liver tissue preconditioning. Additionally, pretreatment of MSCs with injured liver tissue increased their glycogen storage capacity. Transplantation of pretreated MSCs improved their localization and differentiation ability in liver fibrosis mouse models, reducing liver fibrosis and improving liver function. After engraftment, pretreated MSCs showed elevated marker expression for hepatocyte differentiation [159].

### 5.3. MSC Spheroids

The traditional two-dimensional (2D) culture of MSCs exhibits several shortcomings in treating liver diseases, including large-scale expansion of cells, poor survival *in vivo*, and the loss of original properties [160]. Spheroid culture is a novel 3D culture method that preserves natural characteristics such as stemness and secretion, facilitates cell-to-cell and cell-to-matrix communication, creates an in vivo-like growth microenvironment, and improves survival and proliferation [161]. Numerous attempts at preparing 3D-cultured MSCs show advantages in treating end-stage liver disease. Collagen fiber-based 3D spheroids of ADMSCs preserved cell function and paracrine secretion capacity. In addition, transplantation of 3D ADMSCs spheroids alleviated TAA-induced liver cirrhosis in mice [70]. Human-exfoliated deciduous teeth (EDT) are another promising source of MSCs. Hepatocyte-like cells derived from human EDT-MSCs formed spheroids without a scaffold and were transplanted into the livers of mice with CCl4-induced liver fibrosis, where they engrafted, improved liver function, and demonstrated antifibrosis efficacy in mice [162]. Conditioned medium derived from 3D spheroid-derived ADMSCs protected hepatocytes from CCl4-induced injury in vitro by inhibiting hepatocyte apoptosis and LDH release. Furthermore, 3D spheroid-derived ADMSCs improved liver function and reduced liver fibrosis in mice with hepatic fibrosis [163].

### 5.4. Current Challenges and Future Perspectives

The role of MSCs in end-stage liver disease control has been studied in recent years, and substantial progress has been made in understanding how MSCs improve liver diseases. Besides, numerous attempts have been made to enhance the therapeutic effects of MSCs. However, several challenges remain to improving the efficacy of MSCs in the treatment of liver diseases and increasing clinical application.

The consistency, quality control, and sufficient quantity have always been challenges for MSCs in clinical trials and medication development. MSCs come from various origins with unique biological characteristics. Consequently, it is difficult to standardize MSC production and MSC medication [164]. For the treatment of end-stage liver disease, different sources of MSCs should be compared in immunomodulatory, antifibrotic, and liver regenerative capacity to create the optimal treatment strategy. iPSC-MSCs can resolve the issues of inconsistent quality, challenging preparation, and insufficient quantity [38].

The MSC-mitochondrial transfer is one of the benefits of MSCs in treating liver diseases. However, the detailed mechanisms involved have not been sufficiently investigated. Mitochondrial dynamics take part in regulating MSC functions. Some key molecules, including mitofusin 1 and mitofusin 2 (MFN 1 and MFN 2, respectively) [165], optic atrophy 1 (OPA1), and dynamin-related protein 1 (DRP1) regulate MSC dynamics. Posttranslational modifications have also been reported in regulating mitochondria dynamics [166, 167]. The knockout of the key molecules downregulated mitochondria dynamics of MSCs [168]. Certain bioactive compounds enhanced the mitochondrial dynamics and functions of MSCs and increased the efficiency of MSCs [169]. Additionally, 2D and 3D MSC cultures influenced mitochondria dynamics and immunomodulatory functions of MSCs [170]. It is necessary to clarify whether increasing mitochondrial dynamics enhance MSCs in the treatment of end-stage liver disease.

Although MSCs have been widely tested in preclinical and clinical studies of end-stage liver disorders such as liver fibrosis and cirrhosis, there are various issues that must be addressed to improve MSC-based therapy. The pathogenesis of liver fibrosis is a complicated process, and the immunological milieu differs at various phases of pathogenesis. The course of liver fibrosis and the timing of MSC injections must be evaluated.

In a clinical trial, only after patients were infused with UCMSCs for 13 months did survival rates increase [111]. However, further research is needed to determine the precise mechanism by which UCMSC treatment improves survival only after 13 months of treatment.

The ineffectiveness of MSC homing to the injury site is a problem we are facing. Several attempts have been made to enhance MSC homing ability, such as overexpressing CXCR4 or CXCR7 in MSC, which are sensitive to SDF-1. Though the establishment of the chemokine/chemokine receptor axis can help solve this problem in animal models, its safety and efficacy are needed to be investigated in clinical trials [171, 172].

## Figures and Tables

**Table 1:** Clinical trial articles of MSCs in the treatment of liver cirrhosis in PubMed.

Types of liver cirrhosis	Resource of MSC	Dose and passage	Administration times and method	Effect	Country	Reference
HBV-related decompensated liver cirrhosis	UC	0.5 × 10^6^/kg body weight The fourth passage	Intravenously three times at 4-week intervals	Well tolerated, significantly improves long-term survival rate, and the liver function	China	[111]
HBV-related decompensated liver cirrhosis	UC	0.5 × 10^6^/kg body weight The fourth passage	Intravenously three times at 4-week intervals	Well tolerated, a significant reduction in the volume of ascites, and improves liver function	China	[112]
Liver cirrhosis caused by autoimmune diseases	UC, CB, BM	1 × 10^6^/kg body weight The second and fifth passage	Peripheral vein one time	Well tolerated, alanine transaminase values decreased without statistical significance, total bilirubin decreased, serum albumin levels improved, a lowering of prothrombin time, and MELD score improved	China	[113]
Decompensated liver cirrhosis	BM	3.173 × 10^7^ mean value —	Peripheral vein once	Well tolerated, MELD score improved, the quality of life improved	Iran	[114]
Decompensated liver cirrhosis	BM	1.95 × 10^8^ median value Third or fourth passage	Cubital vein once	No beneficial effect	Iran	[115]
HBV, HCV, and alcohol-related liver cirrhosis or cryptogenic liver cirrhosis	BM	3 × 10^7^ − 5 × 10^7^	Portal vein/peripheral vein once	Well tolerated, improved liver function, decreased creatinine, increased serum albumin	Sweden	[116]
Liver cirrhosis	Adipose	1 × 10^8^ —	Intrahepatic injection once	Well tolerated, improved liver function, increased METAVIR score, Child-Pugh score and MELD score, enhanced quality of life	China	[117]
Ursodeoxycholic acid- (UDCA-) resistant primary biliary cirrhosis	BM	3 − 5 × 10^5^/kg body weight The third to fifth passage	Intravenously once	Well tolerated, the life quality and liver function improved, CD8+ T cells reduced, CD4+ CD25+ Foxp3+ T cells increased, serum IL-10 elevated	China	[57]
Alcoholic cirrhosis	BM	5 × 10^7^ —	Femoral artery to hepatic artery one time or twice at 30-day intervals	Well tolerated, collagen reduced, Child-Pugh score improved	South Korea	[118]
Primary biliary cirrhosis with an incomplete response to UDCA	UC	0.5 × 10^6^/kg body weight The fourth passage	Peripheral vein three times at 4-week intervals	Well tolerated, fatigue and pruritus obviously alleviated, liver function improved	China	[119]
HCV-related liver cirrhosis	BM	1 × 10^6^/kg body weight BM-derived undifferentiated and differentiated MSCs —	Intravenously once	Liver function improved	Egypt	[120]
HBV-related liver cirrhosis	BM	— —	Femoral artery to hepatic artery once	Liver function improved, Treg cells significantly increased, Th17 cells markedly decreased, mRNA levels of Treg-related transcription factor (Foxp3) and Th17-related transcription factor (ROR*γ*t) increased and decreased, serum TGF-*β* increased at early times of transplantation, serum IL-17, TNF-*α*, IL-6 reduced	China	[58]
HCV-related end-stage liver diseases	BM	1 × 10^6^^/^kg body weight —	Intravenously once	Liver function improved, collagen decreased without statistical significance	Egypt	[121]
Alcoholic liver cirrhosis	BM	5 × 10^7^ —	Hepatic artery twice at 4-week intervals	Well tolerated, histological improvement, the Child-Pugh score improved, TGF-*β*1, type I collagen and *α*-SMA decreased	Korea	[122]
HBV-related decompensated liver cirrhosis	UC	4.0 − 4.5 × 10^8^ The second passage	Intravenously at the third day and fourth day after conventional treatment	Well tolerated, IL-6, TNF-*α*, and TGF-*β* increased, IL-10 increased, CD4+ T cells and regulatory T cells elevated, CD8+ T cells and B cells reduced, liver function improved, MELD and Child-Pugh scores improved, mortality rate downregulated	China	[27]

**Table 2:** Clinical trials registered at *https://clinicaltrials.gov* of MSCs in the treatment of the chronic end-stage liver diseases (of the approximately 1,348 clinical trials for liver fibrosis/cirrhosis, 68 were MSC-based cell therapy clinical trials; the data comes from *https://clinicaltrials.gov* and the top 29 most recent studies are listed with the highest correlation).

NCT number	Title	Status	Conditions	Interventions	Phase
NCT01220492	Umbilical Cord Mesenchymal Stem Cells for Patients with Liver Cirrhosis	Completed	Liver cirrhosis	(i) Drug: conventional plus UCMSC treatment (ii) Drug: conventional plus placebo treatment	Phase 1 Phase 2
NCT03529136	Clinical Trial of Umbilical Cord Mesenchymal Stem Cell Transfusion in Decompensated Liver Cirrhosis	Unknown	Decompensated liver cirrhosis	Biological: UCMSCs	Phase 2
NCT03254758	A Study of ADR-001 in Patients with Liver Cirrhosis	Recruiting	Decompensated liver cirrhosis	Biological: ADMSCs	Phase 1 Phase 2
NCT03626090	Mesenchymal Stem Cell Therapy for Liver Cirrhosis	Recruiting	Liver cirrhosis	Biological: autologous BMMSCs	Phase 1 Phase 2
NCT02786017	Injectable Collagen Scaffold™ Combined with HUC-MSCs Transplantation for Patients with Decompensated Cirrhosis	Unknown	Decompensated liver cirrhosis	(i) Biological: conventional therapy (ii) Biological: injectable collagen scaffold+human UCMSCs	Phase 1 Phase 2
NCT03460795	Safety and Efficacy Study of Cotransferring of Mesenchymal Stem Cell and Regulatory T Cells in Treating End-Stage Liver Disease	Not yet recruiting	Liver cirrhosis	Biological: MSC and regulatory T cells	Phase 1 Phase 2
NCT05080465	Long-Term Follow-up Mesenchymal Stem Cell Therapy for Patients Virus-Related Liver Cirrhosis	Active, not recruiting	Liver cirrhosis	Biological: autologous BMMSCs	Phase 3
NCT03838250	Study to Evaluate Hepatic Artery Injection of Autologous Human Bone Marrow-Derived MSCs in Patients with Alcoholic LC	Recruiting	Alcoholic liver cirrhosis	Biological: Cell gram™ (BMMSCs)	Phase 1
NCT05121870	Treatment With Human Umbilical Cord-Derived Mesenchymal Stem Cells for Decompensated Cirrhosis	Recruiting	Decompensated liver cirrhosis	(i) Biological: UCMSCs (ii) Biological: saline containing 1% human serum albumin (solution without UCMSCs)	Phase 2
NCT03209986	Trial of Mesenchymal Stem Cell Transplantation in Decompensated Liver Cirrhosis	Unknown	Liver cirrhosis	(i) Procedure: MSC transplantation via peripheral vein (ii) Other: MSCs	Not applicable
NCT04243681	Combination of Autologous MSC and HSC Infusion in Patients with Decompensated Cirrhosis	Completed	Liver cirrhosis	(i) Combination product: CD34+ cells and MSC infusion (ii) Drug: standard of care for cirrhosis management	Phase 4
NCT01342250	Human Umbilical Cord Mesenchymal Stem Cells Transplantation for Patients with Decompensated Liver Cirrhosis	Completed	Liver cirrhosis	(i) Biological: conventional therapy plus low-dose human UCMSC treatment (ii) Biological: conventional therapy plus medium-dose human UCMSC treatment (iii) Biological: conventional therapy plus high-dose human UCMSC treatment	Phase 1 Phase 2
NCT03945487	Mesenchymal Stem Cells Treatment for Decompensated Liver Cirrhosis	Recruiting	Decompensated liver cirrhosis	(i) Biological: UCMSCs (ii) Other: comprehensive treatment	Phase 2
NCT05106972	Umbilical Cord Mesenchymal Stem Cell Transplantation for Decompensated Hepatitis B Cirrhosis	Recruiting	Liver cirrhosis	Drug: UCMSC infusion	Not applicable
NCT01233102	Mesenchymal Stem Cells Treat Liver Cirrhosis	Suspended	Liver cirrhosis	(i) Drug: conserved therapy (ii) Procedure: hepatic artery UCMSC infusion or intravenous infusion	Phase 1 Phase 2
NCT04522869	Umbilical Cord Derived Mesenchymal Stem Cell (UC-MSC) Transplantation for Children Suffering from Biliary Atresia	Recruiting	Primary biliary cirrhosis	Biological: UCMSC transplantation	Phase 1 Phase 2
NCT04357600	Umbilical Cord Mesenchymal Stem Cell for Liver Cirrhosis Patient Caused by Hepatitis B	Recruiting	Liver cirrhosis	Biological: allogeneic UCMSCs	Phase 1 Phase 2
NCT01454336	Transplantation of Autologous Mesenchymal Stem Cell in Decompensate Cirrhotic Patients with Pioglitazone	Completed	Liver fibrosis	Biological: MSC injection	Phase 1
NCT05331872	Umbilical Cord-Derived Mesenchymal Stem Cell Infusion in the Management of Adult Liver Cirrhosis	Recruiting	Liver cirrhosis	Biological: human UCMSC infusion	Phase 1
NCT05227846	Human Umbilical Cord-Derived Mesenchymal Stem Cells for Decompensated Cirrhosis (MSC-DLC-1)	Not yet recruiting	Decompensated liver cirrhosis	Biological: human UCSMCs	Phase 1
NCT05224960	Human Umbilical Cord-Derived Mesenchymal Stem Cells for Decompensated Cirrhosis (MSC-DLC-2)	Not yet recruiting	Decompensated liver cirrhosis	(i) Biological: UCMSCs (ii) Biological: placebo (solution without UCMSCs)	Phase 2
NCT02943889	Stem Cell Transplantation in Cirrhotic Patients	Unknown	Liver cirrhosis	Biological: MSC transplantation	Phase 1 Phase 2
NCT02705742	Mesenchymal Stem Cells Transplantation for Liver Cirrhosis Due to HCV Hepatitis	Unknown	Liver cirrhosis	Biological: autologous ADMSCs	Phase 1 Phase 2
NCT02652351	Human Umbilical Cord-Mesenchymal Stem Cells for Hepatic Cirrhosis	Unknown	Liver cirrhosis	Biological: human UCMSCs	Phase 1
NCT01728727	Safety and Efficacy of Human Umbilical Cord-Derived Mesenchymal Stem Cells for Treatment of HBV-Related Liver Cirrhosis	Unknown	(i) Liver cirrhosis (ii) End-stage liver diseases	(i) Other: UCMSC transplantation (ii) Other: conventional treatment	Phase 1 Phase 2
NCT01662973	Umbilical Cord Mesenchymal Stem Cells for Patients with Primary Biliary Cirrhosis	Unknown	Primary biliary cirrhosis	(i) Other: conventional plus UCMSC treatment (ii) Other: conventional plus placebo treatment	Phase 1 Phase 2
NCT01741090	The Effectiveness and Safety for Mesenchymal Stem Cell for Alcoholic Liver Cirrhosis	Unknown	Alcoholic liver cirrhosis	Biological: BMMSC injection	Phase 2
NCT01483248	Human Menstrual Blood-Derived Mesenchymal Stem Cells for Patients with Liver Cirrhosis	Unknown	(i) Liver cirrhosis (ii) Liver fibrosis (iii) Liver diseases (iv) Digestive system diseases	(i) Biological: conventional therapy plus MSC transplantation (ii) Drug: conventional therapy plus placebo treatment	Phase 1 Phase 2
NCT01440309	Efficacy and Safety Study of Allogenic Mesenchymal Stem Cells for Patients with Refractory Primary Biliary Cirrhosis	Unknown	Primary biliary cirrhosis	(i) Biological: BMMSC (ii) Drug: ursodeoxycholic acid	Phase 1

## Data Availability

All the data is available at https://clinicaltrials.gov and PubMed database.

## Abbreviations

MSCs: Mesenchymal stem cells
HSCs: Hepatic stellate cells
HCC: Hepatocellular carcinoma
PSC: Pluripotent stem cell
BMMSCs: Bone marrow-derived MSCs
UCMSCs: Umbilical cord-derived MSCs
UCPV: Umbilical cord perivascular
ADMSCs: Adipose-derived MSCs
iPSC-MSCs: Induced pluripotent stem cell-derived MSCs
EVs: Extracellular vesicles
Exs: Exosomes
MSC-Exs: MSC-derived Exs
HUCMSC-Exs: Exs derived from human UCMSCs
BMMSC-Exs: BMMSC-derived Exs
HGF: Hepatocyte growth factor
NGF: Nerve growth factor
EGF: Epidermal growth factor
TGF: Transforming growth factor
IGF-1: Insulin-like growth factor-1
Bcl-xl: B cell leukemia-xl
MFGE8: Milk fat globule-EGF factor 8
3D: Three-dimensional
DCs: Dendritic cells
NK cells: Natural killer (NK) cells
IDO: Indoleamine 2,3-dioxygenase
MSC-CM: MSC-conditioned medium
PBC: Primary biliary cirrhosis (PBC)
ECM: Extracellular matrix
LPO: Lipid peroxidation
TMSCs: Tonsil-derived MSCs
GVHD: Graft-versus-host disease
MMPs: Matrix metalloproteinases
EPO: Erythropoietin
FGFs: Fibroblast growth factors
HPCs: Hepatic progenitor cells
HNF-4*α*: Hepatocyte nuclear factor-4*α*
HFMSCs: Hair follicle-derived MSCs
MT: Melatonin
MT-BMMSCs: MT-pretreated BMMSCs
Vit E-WJMSCs: Vitamin E pretreatment of Wharton's jelly-derived MSCs
GSH-ADMSCs/MT-ADMSCs: GSH or MT pretreatment of ADMSCs
ICA: Icariin
2D: Two-dimensional
EDT: Exfoliated deciduous teeth
EDT-MSCs: Exfoliated deciduous teeth-derived MSCs.
